# Sweet Forbearance

**Published:** 1878-11

**Authors:** 


### HALL'S JOURNAL OF HEALTH.

Our Legitimate Scope is almost boundless: for whatever begets pleasurable and harmless feelings, promotes Health; and whatever induces disagreeable sensations, engenders Disease.

WE AIM TO SHOW HOW DISEASE MAY BE AVOIDED, AND THAT IT IS BEST, WHEN SICKNESS COMES, TO TAKE NO MEDICINE WITHOUT CONSULTING A PHYSICIAN.

Vol. 2 ] NOVEMBER, 1878 [No. 11.

### SWEET FORBEARANCE

ONE day, of a past century, at high noon, in Summer, a young man laid by the wayside, in a state of beastly intoxication. The hot sun shone full on his face, and seemed to be burning it to a blister. One and another came and looked, and passed along in silent pity, or cold indifference. At length, a young lady hastily spread her handkerchief over his face, as a partial protection, and was soon lost in the crowd.

When the castaway awoke, partially sobered, he discovered the handkerchief, and on it, the name of one whom he had known in his better days, but who, since he had abandoned himself to his appetites, had refused to acknowledge him as a friend, or even to recognize him as an acquaintance.

The young man read, in this unpretentious act of a young lady whom he had highly esteemed (and with a reciprocity) in his better days, a lesson of sympathy, of reproof, of a quiet exhibition of a forgiving nature, with a willingness to do what she might to bring him back to his former self, and he resolved, then and there, that he would never "drink another drop" again. And by the grace of God, and a manly determination, he never did! His reform was radical; his attentions were renewed, and after a season sufficiently long to certify to the earnestness of his purpose, the young lady married WILLIAM WIRT, who subsequently became one of the purest, and best, and brightest men of a past generation.

Many a young woman is there, who, in the loftiness of her own virtue, and in the stately pride of an untried heart, would have passed such an erring brother by in indifference, contempt, or in deeper disgust, and thus have left him to become lost to himself, to his country, and to the world.

Vice we may rightly abhor; drunkenness we may, and ought to despise; but the vicious and the drunken need our sympathies, and strongly claim our countenance—we should even go out of our way, in our efforts to lift them on their feet again, to hold them up, and help them forward, as long as they are willing to try and take a step themselves in the right direction.

Many are there who would have passed by, doing nothing then, only afterwards to busy themselves in thoughtless, if not in malicious rehearsals of what they had seen, forgetting the deep reprobation of conduct so inexcusable.

Let it be remembered, that a true humanity consists in sympathy for the erring—a sympathy not theoretical and superficial, but practical and real; a sympathy which spends itself in words of kindness, and in acts of love and help. Let us remember, that it is not for man, the creature worm, to sit as judge and executioner of his brother man. Let justice and judgment be in the hands of the Great Father of all. The province of the creature is to live in the constant practice of the warm benevolences of a better and a higher nature; for it is by these, and these mainly, that we are to throw around this world a chain of love, and raise it up to God! This is Christ-like—this is divine.

